# *Trichinella spiralis* and *T. britovi* in North-Eastern Romania: A Six-Year Retrospective Multicentric Survey

**DOI:** 10.3390/vetsci9090509

**Published:** 2022-09-17

**Authors:** Olimpia Iacob, Ciprian Chiruță, Mihai Mareș

**Affiliations:** 1 Department of Clinics, Parasitology and Parasitic Diseases, Faculty of Veterinary Medicine, “Ion Ionescu de la Brad” University of Life Sciences, 700489 Iași, Romania; 2 Department of Sciences, Faculty of Horticulture, “Ion Ionescu de la Brad” University of Life Sciences, 700489 Iași, Romania; 3 Department of Public Health, Faculty of Veterinary Medicine, “Ion Ionescu de la Brad” University of Life Sciences, 700489 Iași, Romania

**Keywords:** *Trichinella spiralis*, *T. britovi*, prevalence, pig, horse, wild boar, bear, northeast Romania

## Abstract

**Simple Summary:**

The genus *Trichinella* includes roundworm parasites with a wide geographical spread that can cause illness in humans and animals. In this context, an epidemiological study of *Trichinella* infection was carried out in the northeastern part of Romania to investigate for the first time its prevalence in pigs, horses, wild boars and bears, the geographical distribution of *Trichinella* species and the natural reservoir of the parasites. Between 2010 and 2015, a total of 166,270 animals were examined by specific methods in order to calculate the prevalence of *Trichinella* infection, the involved species, and their geographical distribution. The overall prevalence of *Trichinella* infection in animals was 0.188%. But the specific prevalence varied as follows: in pigs 0.096%, horses 0.021%, wild boar 1.46% and bears 36.76%. The geographical distribution showed that *T. spiralis* was dominant, occupying the entire northeastern part of Romania, being identified in pigs, horses, wild boars and bears. *T britovi* occupied five mountain counties, being identified only in wild boars and bears. These results validate the presence of *T. spiralis* and *T. britovi* in domestic and game animals in northeast Romania and warn about the risk of human infection in the region.

**Abstract:**

The genus *Trichinella* includes species with a wide geographical spread that cause pathology in humans and animals. In this context, an epidemiological study of *Trichinella* infection was carried out in the northeastern part of Romania to investigate for the first time the prevalence of this infection in pigs, horses, wild boars and bears, the geographical distribution of *Trichinella* species and the natural reservoir of *Trichinella* infection. Between 2010 and 2015, a total of 166,270 animals were examined by the method of artificial digestion, in order to calculate the annual and general prevalence of *Trichinella* infection, according to the host and the *Trichinella* species involved, the Pearson correlation coefficient (r), trendline and geographical distribution of species of the genus *Trichinella*. Taxonomic framing was performed by the multiplex PCR method. The overall prevalence of *Trichinella* infection in animals was 0.188%. Within the host species, the prevalence varied as follows: in pigs 0.096%, horses 0.021%, wild boar 1.46% and bears 36.76%. The geographical distribution showed that *T. spiralis* was dominant, occupying the entire northeastern part of Romania, being identified in pigs, horses, wild boars and bears. *T britovi* occupied five mountain counties, being identified only in wild boars and bears. These results validate the presence of *T. spiralis* and *T. britovi* in domestic and game animals in the northeastern part of Romania.

## 1. Introduction

Trichinellosis is a severe parasitic zoonosis caused by species of the genus *Trichinella* [1], with a wide geographical spread [2], affecting a wide range of hosts (mammals, birds, reptiles) [3]. The geographical distribution of the genus *Trichinella* is influenced by human intervention in the habitat of domestic and wild animals [4]. *Trichinella* spp. belong to the phylum Nematoda, class Enoplea, order Trichocephalida, family Trichinellidae, genus *Trichinella* [5,6]. Currently, the genus *Trichinella* includes 10 species and 3 genotypes divided into two clades: encapsulated species such as *Trichinella spiralis* (T1), *Trichinella nativa* (T2), *Trichinella britovi* (T3), *Trichinella murrelli* (T5), *Trichinella nelsoni* (T7), *Trichinella patagoniensis* (T12), *T. chancalensis* (T13), and three genotypes (*Trichinella* T6, T8 and T9), and non-encapsulated species such as *Trichinella pseudospiralis* (T4), *Trichinella papuae* (T10), *Trichinella zimbabwensis* (T11) [5,7]. In Europe, there are four prevalent species: *T. spiralis*, *T. britovi*, *T. nativa*, and *T. pseudospiralis* [8,9]. *Trichinella spiralis* has the highest prevalence in domestic animals (pig, horse) and is also identified in game (wild boar, bear) [10], whereas *T. britovi* is the more widespread among wild carnivores but also infects domestic and wild pigs [11]. In humans, trichinellosis is transmitted by eating raw or incompletely cooked meat and meat products from domestic animals (pig, horse) and game (wild boar, bear), parasitized with larvae of *Trichinella* spp. [12,13,14,15]. Over time, the social and economic impact of *Trichinella* species has greatly influenced the epidemiological view of this zoonosis [4]. Currently, human population growth and socioeconomic changes have led to people moving to new ecological regions and changes in animal husbandry practices, which could have an impact on the occurrence of trichinellosis in humans and *Trichinella* infections in animals. [16]. The control of trichinellosis, regulated by the normative acts in force (EU regulation 2075/2005, Codex Alimentarius: CAC, 2015; Health Code for terrestrial animals OEI), is rigorously applied in the European Union and is estimated at an annual cost between 25 and 400 million [17]. Previous studies on *Trichinella* infection in animals and the impact of this zoonosis on humans in Romania have been performed by Blaga et al. [18], Iacob and Tășchină-Nicolae [19], Neghină et al. [20] and Nicorescu et al. [21] emphasizing the importance of *Trichinella* infection in domestic and game animals as a source of trichinellosis in humans.

The current paper is an epidemiological study on the prevalence and geographical distribution of *Trichinella* species in domestic animals (pigs and horses) and game (wild boars and bears) in northeastern Romania to elucidate the current *Trichinella* infection status in Romania and become a useful working tool in comparative processing of data by region. 

## 2. Materials and Methods

### 2.1. Epidemiological Study

#### 2.1.1. Geographical Area

The study was based on the analysis of data from 2010 to 2015 provided by the Veterinary and Food Safety Laboratories in all counties in North-East Romania. The area investigated comprises 36,850 km^2^ with a total population of 3,674,367 inhabitants ranging from 44.78–48.24° north latitude and between 28.05–28.24° east longitude, respectively [22]. Geographically, all the natural features are present (plain, plateau, hill, mountain), ensuring a different climate with varied fauna and flora. The counties of Suceava (SV), Neamț (NT), Bacău (BC), Vrancea (VN) and Buzău (BZ) are located in the mountainous area, being populated with wild boars, bears, and other wild carnivores (wolf, fox, lynx, wild cat, etc.). Meanwhile, the counties of Botoșani (BT), Iași (IS), Vaslui (VS), Galați (GL) are located in hilly, plateau and plain areas, being populated with wild boar. The variety of the natural features favors the circulation of wild animals from one area to another, complicating the epidemiological surveillance of *Trichinella* infection. The geographical distribution of the host animals highlights that bears inhabited the territory of five neighboring counties (SV, NT, BC, VN, BZ), and the wild boar inhabited all counties. Pigs raised in an industrial or extensive household system were present in all counties, and horses in three counties (SV, BT and BZ).

#### 2.1.2. Collection and Examination of Samples-Identification of Trichinella Species

During the analyzed period, a total number of 166,270 samples of muscle tissue from domestic animals (pig: 131,759; horse: 23,748) and wild animals (wild boar: 10,695; bear: 68) were examined. They were examined after slaughter in the slaughterhouse and in the households of the population or after collection by shooting during the hunting season. An average sample of 50 g of muscle tissue (diaphragm, intercostal muscles and tongue) was taken from each carcass. The examination of samples was done in specialized laboratories by artificial digestion, according to the protocol developed by the European Commission [23]. Positive cases were sent to the Institute of Veterinary Hygiene and Public Health (IISPV) Bucharest and the European Reference Laboratory in Rome for the molecular identification of Trichinella species. The identification of *Trichinella* species was made by the multiplex PCR method, according to the protocol established by Pozio and La Rosa (2003). Five pairs of primers were used: Primer pair I: 5′GTTCCATGTGAACAGCAGT-3′; 5′-CGAAAACATACGACAACTGC-3′; Primer pair II: 5′-GCTACATCCTTTTGATCTGTT-3′; 5′-AGACACAATATCAACCACAGTACA-3′; Primer pair III: 5′-GCGGAAGGATCATTATCGTGT-3′; 5′-ATGGATTA CAAAGAAAACCATCACT-3′; Primer pair IV: 5′-GTGAGCGTAATAAAGGTGCAG-3′; 5′-TTCATCACACATCTTCCACTA-3′; Primer pair V: 5′-CAATTGAAAACCGCTTAGCGTGTTT-3′; 5′TGATCTGAGGTCGACATTTCC-3′ were designated to amplify the internal transcribed spacers ITS1 and ITS2, and the expansion segment V (ESV) region of the ribosomal DNA. 10 µL of total DNA were subjected to multiplex PCR in a 30 µL mixture reaction. The mix for the detection of the target sequence contained 1× PCR buffer, 3 mM MgCl_2_, 0.2 mM of each deoxynucleotide triphosphate, 0.3 µM of each primer and 1 U of Taq polymerase. Amplification was carried out as follows: initial denaturation at 95 °C for 4 min; 40 cycles of 95 °C for 10 s, 55 °C for 30 s and 72 °C for 30 s; and a final elongation step at 72 °C for 3 min. DNA fragments were analyzed by electrophoresis in a 2% agarose gel in 1× TAE buffer (40 mmol/l Tris–HCl, 2 mmol/l acetate, 1 mmol/lEDTA) and stained with ethidium bromide. The bands in the gel were visualized and photographed under UV light [24].

### 2.2. Statistical Analysis

The statistical analysis was performed using MS EXCEL 2016 software. Confidence intervals (CI) were calculated, and α = 0.05 was considered statistically significant [25]. The annual and general prevalence of *Trichinella* spp. infection in pigs, horses, wild boar and bears was evaluated in each county and cumulatively throughout the northeast, as well as the geographical distribution of *Trichinella* and host species. The Pearson correlation coefficient (R^2^) and the *Trichinella* infection trendline were calculated. The results obtained were framed in tables and represented graphically (trendline and correlation coefficient R^2^) but also distributed in the maps.

## 3. Results

### 3.1. The Prevalence and Dynamics of Trichinella Infection in North-Eastern Romania

The data study reveals that the overall prevalence of *Trichinella* infection in the examined animals was 0.188% (313/166,270 samples).

The prevalence varied depending on the host species as follows: in pigs 0.096% (127/131,759); in horses 0.021% (5/23,748) in wild boar 1.46% (156/10,695) and in bears 36.76% (25/68), (Table 1).

**Figure 1:**
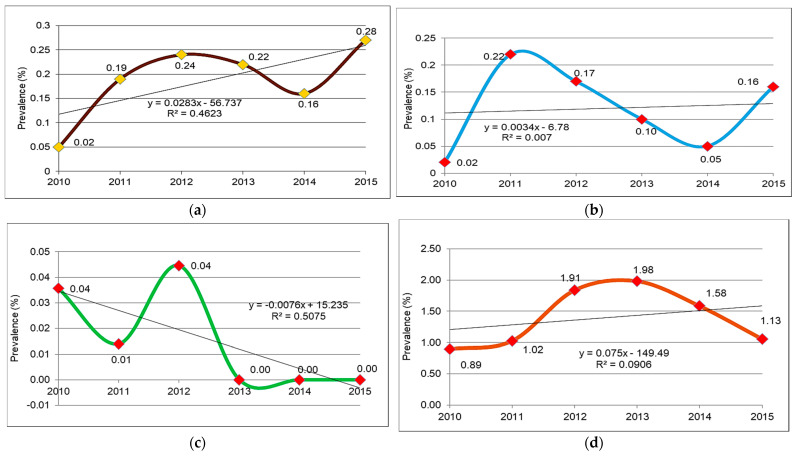

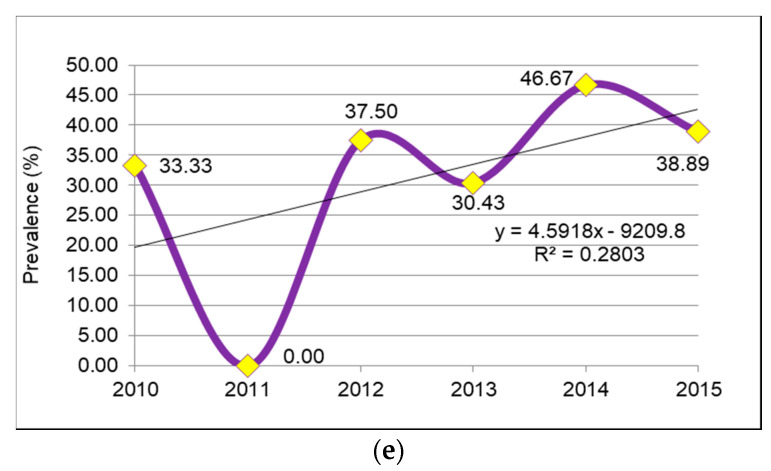
(**a**) Dynamics of the general prevalence (%) of *Trichinella* infection in animals in Northeastern Romania (2010–2015); (**b**) Specific dynamics of *Trichinella* infection in pigs (2010–2015); (**c**) Specific dynamics of *Trichinella* infection in horses (2010–2015); (**d**) Specific dynamics of *Trichinella* infection in wild boar (2010–2015); (**e**) Specific dynamics of *Trichinella* infection in bears (2010–2015).

The dynamics of the general prevalence of *Trichinella* infection, the Pearson correlation coefficient (R^2^) and the trendline are presented in Figure 1a.

The general prevalence of *Trichinella* infection in animals shows close values over the entire period studied (2010–2015), with oscillating dynamics. It started at 0.02% in 2010 and reached a maximum of 0.28% in 2015. In this case, there is an ascending trend, also defined by the line of predictability, and the Pearson correlation coefficient reveals an average correlation (R^2^ = 0.4623). Prevalence values, although low, suggest a persistent *Trichinella* infection in the population of domestic animals (pigs and horses) and wild animals (wild boars and bears) spread throughout the northeast.

The prevalence dynamics, the Pearson correlation coefficient (R^2^) and the trendline are indicated in Figure 1b.

In pigs, the prevalence of *Trichinella* infection indicates minimal values (0.02%) in 2010, an increase (0.22%) in the following year (2011), to subsequently register a descending trend until 2014 (0.05%), with a new trend increase (0.16%) in 2015. In fact, there is a tendency to equalize the predictability line and a very, very weak correlation (R^2^ = 0.007) of the infection. The prevalence of *Trichinella* infection, even with low values, confirms the persistence of parasites in the pig population throughout the analyzed period in northeastern Romania.

The prevalence dynamics of *Trichinella* infection in horses, the Pearson correlation coefficient (R^2^) and the trendline are shown in Figure 1c.

In horses, the specific dynamics of *Trichinella* infection are particular and are due to a small number of cases compared to a large number of animals examined. Thus, in 2010, the prevalence was 0.035%, decreased in the following year (2011) to 0.014% and increased (0.044%) in 2012. Subsequently, it fell steadily over the next three years to 0.00%. The clearly descending aspect of the predictability line and an average correlation (R^2^ = 0.5075) of the infection was noticed.

The prevalence dynamics of *Trichinella* infection in wild boar, the Pearson correlation coefficient (R^2^) and the trendline are demonstrated in Figure 1d.

The dynamics of the prevalence of *Trichinella* infection in wild boars describe a simple line, starting with 0.89% in 2010, reaching a peak of 1.98% in 2013 and decreasing to 1.13% in 2015. In wild boars, the annual value of prevalence was higher (0.89–1.98%) than in pigs and horses, recorded in the same analyzed period. The predictability line of *Trichinella* infection is slightly ascending, and the Pearson correlation coefficient (R^2^ = 0.0906) indicates a very weak correlation of the infection. 

The prevalence dynamics of *Trichinella* infection in bears, the Pearson correlation coefficient (R^2^), and the trendline are indicated in Figure 1e.

In bears, the dynamics of prevalence are sinuous and are positioned on both sides of the predictability line. The annual prevalence has higher values compared to wild boars and oscillates from 0.00% in 2011 to 46.67% in 2014. The predictability line has an ascending aspect, revealing a weak correlation (R^2^ = 0.2803) of the infection. 

### 3.2. Geographical Distribution of Trichinella Infection in Animals in North-Eastern Romania

**Figure 2:**
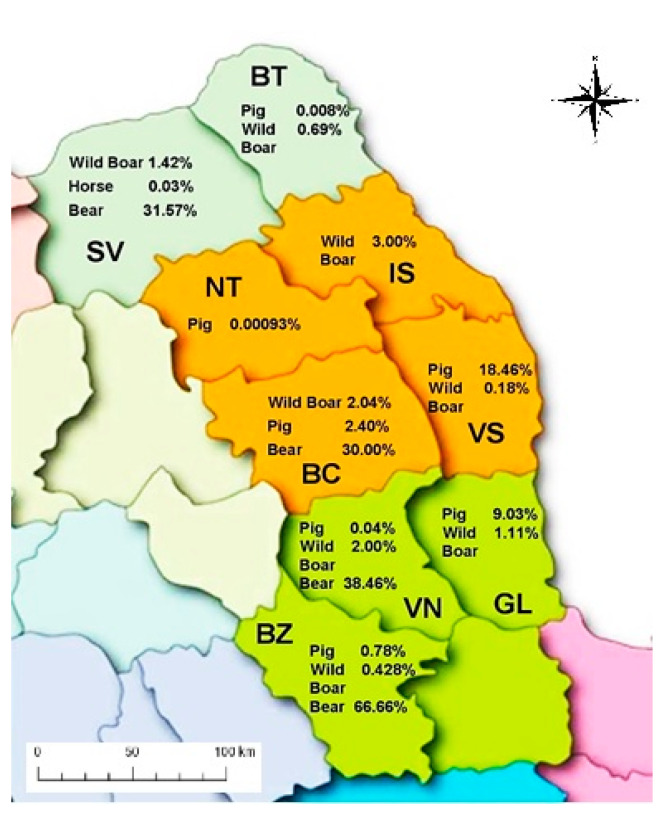
Geographical distribution and prevalence of positive samples for *Trichinella* infection in animals in North-Eastern Romania (2010–2015).

The geographical spread of *Trichinella* infection in northeastern Romania is included in Table 2 and illustrated in Figure 2.

The estimation of the prevalence of infection in geographically distributed animals was made using a confidence interval because it covers the real value of prevalence with a given probability. 

The geographical distribution of *Trichinella* infection in pigs shows that the infection was caused only by *T. spiralis* with a prevalence of 0.096% (127/131,759), but with huge variability. The minimum prevalence of 0.000098% (1/107,675) was found in Neamț county, and the maximum prevalence of 18.46% (12/65) was registered in Vaslui county. The geographical distribution of *Trichinella* infection in horses indicates that the infection was caused only by *T. spiralis* with a general prevalence of 0.021% (5/23,748), ranging from zero in Botoșani (0/5244) and Buzău (0/97) counties to 0.03% (5/18,407) in the Suceava county. The geographical distribution of *Trichinella* infection in wild boar reveals that the infection was caused by *T. spiralis* and *T. britovi*, with a general prevalence of 1.46% (156/10,695), ranging from zero in Neamț (0/211), to 3.00% (15/500) in Iași county, without co-infection. The geographical distribution of *Trichinella* infection in bears illustrates the infection was caused by *T. spiralis* and *T. britovi*, with a prevalence of 36.76% (25/68), ranging from zero (0/3) in Neamț and Galați (0/1) to 66.66% (8/12) in Buzău county, without co-infection.

### 3.3. Geographical Distribution and Prevalence of T. spiralis and T. britovi, in Animals, in North-Eastern Romania

**Figure 3:**
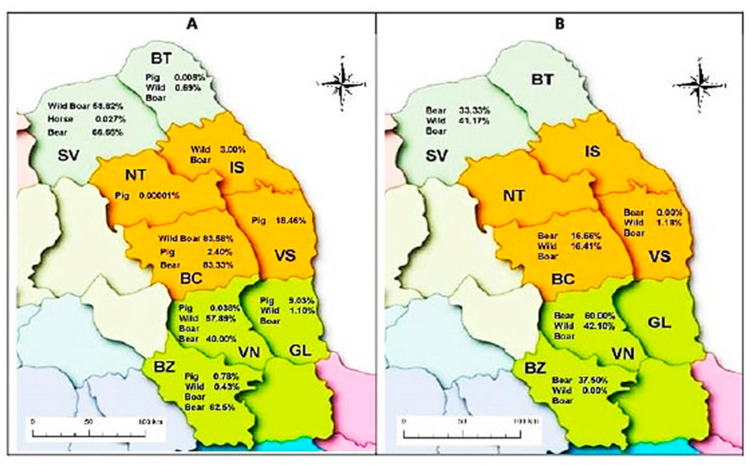
Geographical distribution of positive samples for *Trichinella spiralis* (**A**) and *Trichinella britovi* (**B**).

The geographical distribution of *T. spiralis* and *T. britovi* species is different and unequal and is influenced by a multitude of factors. *T. spiralis* has a wide geographical spread, being identified in all (nine) counties, in pigs, horses, wild boar, and bears, with varying prevalence (Table 3). The geographical distribution of the *T. spiralis* species in North-Eastern Romania is illustrated in Figure 3A.

The data in Table 3 emphasize that *T. spiralis* is geographically widespread in all counties of northeast Romania, with a different prevalence in both synanthropic and sylvanic environments. So, in the synanthropic environment (pigs: 127/131,759 and horses: 5/23,748), the prevalence of *T. spiralis* infection was 0.085% (132/155,507). In the sylvatic environment (wild boar (119/4189) and bears (16/25), *T. spiralis* had a prevalence of 3.20% (135/4218). Within the total number of *Trichinella* spp infections, *T. spiralis* had an overall prevalence of 74.58% (135 *T. spiralis*/181 (156 + 25, Table 1, total *Trichinella* infections), where the prevalence of *T. spiralis* in the wild boar was 76.28% (119 *T. spiralis*/156–total *Trichinella* infections), and in bears was 64% (16 *T. spiralis*/25 total *Trichinella* infections).

*T. britovi* is geographically restricted to the mountainous area, counties SV, BC, VN and BZ, where it has been identified in bears, and counties SV, BC, VN and VS, where it has been identified in wild boars (Table 4). The distribution of *T. britovi* in both bears and wild boars in the same mountain counties (SV, BC, VN) is noticeable, which confirms the sylvatic maintenance of the *Trichinella* infection and a source of infection for other hosts.

The geographical distribution of *T. britovi* species in bear and wild boar is illustrated in Figure 3B.

Within the genus *Trichinella, T britovi* was identified only in the sylvatic environment, in game (bear and wild boar) with a prevalence of 25.41% (46 *T britovi*/181 total game positive (156 wild boar + 25 bears + Table 1). In wild boars, the prevalence of *T. britovi* was 23.72% (37/156), and in bears, it was 36% (9 *T. britovi*/25 total *Trichinella* positive). No co-infections of the two species were reported in either bears or the wild boar. The overall prevalence of *T. britovi* was 0.43% (46 *T. britovi*/10,763 total samples examined, comprising 10,695 wild boars and 68 bears). 

## 4. Discussion

The northeastern part of Romania, historically known as Moldova, includes nine counties arranged from northeast to south, as follows: SV-BT, NT-IS, BC-VS, VN-GL and BZ, defining the distribution area of the host animals and the origin of the samples examined.

The prevalence of *T. spiralis* and *T. britovi* species and the geographical distribution in the North-Eastern part of Romania were influenced by numerous factors, including natural features, forested areas, wild animals, agricultural areas, rural population preference for extensive pig breeding, education and public awareness regarding the veterinary sanitary control of meat obtained from the household or from game meat.

Numerous studies on the prevalence of *Trichinella* infection in animals have been undertaken in Romania’s neighboring countries. In this regard, research conducted by Lalkovski (2017) in Bulgaria during the same period (2010–2016) reveals that *Trichinella* infection was caused by *T. britovi* (94.17%) and *T. spiralis* (5.83%). Both species were identified in pigs and wild boars in a ratio of 45:1 in wild boars and 1:1 in pigs. *Trichinella britovi* was the most widespread geographically, being identified throughout the country, while *T. spiralis* was identified only in a few areas [26]. In Hungary, research conducted by Szell et al. (2012) [15] show that *Trichinella* infection was identified in wild boars, with a very low prevalence of 0.0077%. The species identified were *T. britovi* (64.7%), *T. spiralis* (29.4%) and *T. pseudospiralis* (5.9%), and their geographical distribution shows that the level of risk differs from one area to another.

In a recent study by Klun et al. [27] it was shown that in Serbia, *T. spiralis* had a prevalence of 77.8% in wild carnivores, respectively in red fox and wild cat, and *T. britovi*, in the same hosts, had a prevalence of 22.2%. The predominance of *T. spiralis* in wild animals in Serbia indicates the transition of this species from domestic to wild animals [27].

The geographical distribution of *Trichinella* species in Europe differs from country to country. Thus, *T. spiralis*-the most pathogenic species to humans has an uneven distribution with important foci in Eastern countries [28]. In most countries, *T. britovi* is more widespread (62.5–100%) than *T. spiralis* (0.0–37.5%), although in Finland, Germany, Poland and Spain, *T. spiralis* is more widespread (56.3–84.2%) [29]. In Poland, *Trichinella* infection in animals is caused by *T. spiralis* and *T. britovi* species, but recently Bilska-Zając et al. [12] identified *T. nativa* in wild boar, confirming the spread of this species in new regions of Europe. In Greece, *Trichinella* infection is caused by *T. britovi,* with a prevalence of 0.29% in pigs and 6.4% in wild boars [30]. In Italy, *Trichinella* infections are caused by *T. spiralis* and *T. britovi*-species identified in domestic and wild animals [31]. A case of simultaneous parasitism with both species has been reported in horses [32]. *Trichinella pseudospiralis* is also present in Italy, and this species was reported in two owls (*Strix aluco* and *Athene noctua*), one red kite (*Milvus milvus*), five wild boars (*Sus scrofa*), one wolf (*Canis lupus italicus*), and one red fox (*Vulpes vulpes*) [33]

Prevalence studies conducted by Serrano et al. [34] and Boadella et al. [35] show that in Spain, *Trichinella* infection is caused by *T. britovi* and *T. spiralis* species. In the Extremadura region, *T. britovi* has been found in wild boar in more than a quarter of cases of *Trichinella* infection, with a higher level of infection than *T. spiralis* [34]. In the central part of the country, the average prevalence of *Trichinella* infection in wild boars was 0.2% [35]. Research by Deksne et al. (2016) [36] show that in Latvia, *Trichinella* infection is caused by *T. britovi*, *T. nativa* and *T. spiralis* species, with an overall prevalence of 2.5% in wild boars [11]. *T britovi* had a maximum prevalence of 94.0%; native *Trichinella* was detected in single (1.1%) or mixed (4.4%) infection with *T. britovi*; *T. spiralis* has been detected in mixed infection with *T. britovi* [36]. 

Regarding the environmental conditions, there is no difference between the two species of *Trichinella*, although *T. britovi* prefers habitats at higher altitudes than *T. spiralis* [29]. Some studies show that *T. spiralis* (T1) has the highest prevalence (43.3%) of all species and genotypes of the genus *Trichinella*, followed by *T. britovi* (T3) (41.2%) [37]. Other studies show that the species *T. britovi* (T3) has a higher prevalence (44.8%) compared to *T. spiralis* (T1), 39.9% [4]. The two species dispute their primacy according to numerous factors, including identification methods. The combined use of serological ELISA and Western blot methods is 31.4 times more sensitive than digestion (32/1462 vs. 1/1462), suggesting their potential use for epidemiological surveillance of *Trichinella* infection in wild boar populations and other host animals [13,38].

Wild animals are the most important reservoir for the genus *Trichinella* and an important source of infection for domestic animals and humans [39,40]. The high prevalence of *Trichinella* spp. in wildlife suggests that they are indicators for assessing the risk of infection with *Trichinella* spp. [36]. 

In Romania, Blaga et al. [18] reported similar values of the prevalence of *T. spiralis* species (49.2%), compared to *T. britovi* (50.8%), due to the numerous household outbreaks associated with pig herds.

Nicorescu et al. [21] conducted an epidemiological study on the prevalence of *Trichinella* spp. in pigs, wild boars and bears throughout Romania, reporting that in bears, the prevalence was highest (12.93%), followed by wild boar (1.66%) and pigs (0.20%). Multiplex PCR analysis of *Trichinella*-positive isolates revealed that *T. spiralis* had a prevalence of 74.49% compared to 22.45% in *T. britovi*; the mixed infection with the two *Trichinella* species was 3.0%. The authors reported that *Trichinella* infections were widespread in all areas but with a different prevalence. Thus, in the south and southeast of Romania, *T. spiralis* was identified at 98.25% and 87.88%, respectively, compared to *T. britovi*, identified at 1.75% and 12.12%, respectively. The same authors show that in the North-East, the prevalence of *Trichinella* infection was 2.8% in game and 0.01% in pigs. In the northwest, the *Trichinella* infection was 2.48% in game and 1.52% in pigs. The authors note that, geographically, *T. spiralis* covers the entire territory of Romania, being identified in pigs and game, while *T. britovi* was present in game in all areas, and in pigs, only in the central, southwestern, and northwest areas [21]. Our study confirms the data communicated by Nicorescu et al. by identifying *T. spiralis* in pigs, wild boar, bears, and in addition, in horses, while *T. britovi* was identified only in wild boar and bear, with a very different prevalence from one species to another. 

From studies on *Trichinella* infection in animals, it is observed that in European countries, the prevalence values are very different, being either in favor of *T. spiralis* or in favor of *T. britovi*, without being a common regulatory-equalizing factor. Each country or area has its own specifics, including climate factors, relief, vegetation, the presence of forests, agricultural areas, domestic animals, wildlife and the human population with traditions, customs, level of culture and civilization.

Thus, the prevalence of *Trichinella* infection in animals is close in value in some areas and very different in others. There is no uniformity in the presence, dynamics and distribution of *Trichinella* infection in animals. Our epidemiological study covers a Romanian geographical area that, until now, has been studied only partially and never in its entirety as part of the northeast. 

From this point of view, our study demonstrates that, in the northeastern part of Romania, *T. spiralis* is present in all counties, being identified in all examined animal species. The highest prevalence was in game (wild boar: 83.58%; bears: 83.33%), followed by domestic animals (pigs: 18.46% and horses: 0.027%). 

*T. britovi* was geographically present in five mountain counties, identified only in game, with the maximum prevalence in bears (60.00%), followed by wild boar (42.10%). The mountainous relief and the forested areas with different altitudes offer favorable conditions for the bear and wild boar population development. This aspect is present in three counties SV, BC and VN (Table 4), where bears and wild boars coexist, contributing to developing and maintaining a natural reservoir of *Trichinella* spp. The number of bears and wild boars is regulated in the hunting season, but in abundant feeding conditions, wild boar populations grow much faster, exceeding the ability of hunters to regulate the number of individuals in a forest area. In hilly areas, with deciduous forests and sufficient food, abundant populations of invading wild boars and agricultural regions are developing, causing economic damage. Our results are consistent with data from the literature [18,20,21,41]. 

In humans, a retrospective analysis of trichinellosis shows that worldwide, between 1986 and 2009, 65,818 cases and 42 deaths were reported in 41 countries, a context in which Europe accounted for 86% of cases (56,912). Of these cases, 28,564 (50%) were reported in Romania between 1990 and 1999 [40,42]. However, in the last 16 years (from 2002 to 2017), in the European Union, there has been a decrease in the incidence of trichinellosis, with 5518 cases reported. However, Bulgaria and Romania reported, in 2017, more than half of the confirmed cases and outbreaks [40].

According to data reported by the National Institute of Public Health (INSP) and the National Center for Surveillance and Control of Communicable Diseases (CNSCBT) in Romania, between 2010 and 2017, human trichinellosis showed a variable incidence per 100,000 inhabitants. Thus, the incidence was 0.9%_000_ in 2010; 0.7%_000_ in 2011; 1.3%_000_ in 2012; 0.94%_000_ in 2013; 1.61%_000_ in 2014; 0.48%_000_ in 2015; 0.44%_000_, in 2016 and 0.69%_000_, in 2017, suggesting a downward trend of infection among the population. The annual fluctuation in the number of cases is probably the consequence of the consumption of pork and wild boar products during the winter holidays, during the hunting season, the culinary habits, the tradition of preparing meat dishes, the consumption of raw dishes and the lack of veterinary examination [43].

It is known that humans become infected with all species of the genus *Trichinella*, but *T. spiralis* is the most pathogenic to humans. In most cases, infection is manifested by allergic reactions (facial edema), muscle pain, gastrointestinal disorders, heart disorders, and non-specific clinical signs which develop variously, sometimes fatally, depending on different factors such as the source of infection and the number of larvae ingested [29].

Trichinellosis is continuously reported in humans in Romania. Therefore, the assessment and monitoring of risk factors should be improved in both domestic and game animals and other wild species to monitor the presence and prevalence of these parasites [41].

Sustained epidemiological surveillance in the prevention of trichinellosis in humans leads to a decrease in the incidence and impact of this disease on the population’s health [30]. Important elements of this activity include the allocation of economic funds, the improvement of animal husbandry practices, meat inspection, consumer education, medical care and the constant updating of prophylaxis measures [43].

## 5. Conclusions

*T. spiralis* was found prevalent in North-Eastern Romania, being present in all nine counties and in all hosts examined, which included pigs, horses, wild boars, and bears, with a general prevalence of 0.18% (283/159,750). *T. britovi* was dominant in wild boar and bear, being present in five counties, with a general prevalence of 0.43% (46/10,763). No mixed infections with the two species were reported in the same host animal. The prevalence of *Trichinella* infection in the North-Eastern part of Romania, particularly in the game animals, confirms the presence of a well-preserved sylvatic reservoir of the parasite. This, constitutes a greater risk of infection to humans and of dispersion to synanthropic animals, suggesting increased attention should be paid to consumers that occasionally purchase meat or meat products without veterinary examination.

## Figures and Tables

**Table 1:** The annual and general prevalence of *Trichinella* infection in animals from North-Eastern Romania.

Year	Total Samples Examined		Pig		Horse		Wild Boar		Bear	
	Positive/ Tested	C.I. [a–b]	Positive/ Tested	C.I. [a–b]	Positive/ Tested	C.I. [a–b]	Positive/ Tested	C.I. [a–b]	Positive/ Tested	C.I. [a–b]
2010	17/30,645	0.0476–0.0524	7/26,939	0.0183–0.0217	1/2807	0.0282–0.0418	8/896	0.2750–1.5050	1/3	0.0000–86.6731
2011	32/16,615	0.1840–0.1960	18/8200	0.2110–0.2290	1/7144	0.0113–0.0167	13/1270	0.4674–1.5726	0/1	0.0000–0.0000
2012	54/22,240	0.2344–0.2456	24/14,248	0.1638–0.1762	3/6733	0.0391–0.0489	24/1251	1.1515–2.6685	3/8	3.9520–71.0480
2013	76/34,036	0.2156–0.2244	25/26,093	0.0964–0.1036	0/5702	0.0000–0.0000	44/2218	1.4002–2.5598	7/23	11.6258–49.2342
2014	59/36,542	0.1562–0.1638	18/33,770	0.0477–0.0523	0/610	0.0000–0.0000	34/2147	1.0525–2.1075	7/15	21.4227–71.9173
2015	75/26,192	0.2746–0.2854	35/22,509	0.1552–0.1648	0/752	0.0000–0.0000	33/2913	0.7462–1.5138	7/18	16.3686–61.4114
Total	313/16,6270	0.1861–0.1899	127/131,759	0.0944–0.0976	5/23,748	0.0192–0.0228	156/10,695	1.2327–1.6873	25/68	25.3000–48.2200
General prevalence (%): 0.188			0.096		0.021		1.46		36.76	

**Table 2:** Geographical distribution of *Trichinella* infection in animals in North-Eastern Romania.

County	Pig		Horse		Wild Boar		Bear	
	Positive/ Tested	C.I. [a–b]	Positive/ Tested	C.I. [a–b]	Positive/ Tested	C.I. [a–b]	Positive/ Tested	C.I. [a–b]
Suceava (SV)	0/1109	0.0000–0.0000	5/18,407	0.0248–0.0295	34/2397	0.9450–1.8918	6/19	10.6777–52.4802
Botoșani (BT)	1/12,308	0.0065–0.0097	0/5244	0.00000–0.00000	1/145	0.0000–0.7650	0	0.0000–0.0000
Neamț (NT)	1/107,675	0.0007–0.0011	0/0	0.00000–0.00000	0/211	0.0000–0.0000	0/3	0.0000–0.0000
Iași (IS)	0/183	0.0000–0.0000	0/0	0.00000–0.00000	15/500	1.5047–4.4953	0	0.0000–0.0000
Bacău (BC)	3/125	0.0000–5.0831	0/0	0.00000–0.00000	67/3279	1.5591–2.5276	6/20	9.9160–50.0840
Vaslui (VS)	12/65	9.0293–27.8938	0/0	0.00000–0.00000	4/338	0.0306–2.3363	0	0.0000–0.0000
Vrancea (VN)	3/7825	0.0341–0.0426	0/0	0.00000–0.00000	19/950	1.1097–2.8903	5/13	12.0149–64.9082
Galați (GL)	96/1063	7.3080–10.7541	0/0	0.00000–0.00000	6/542	0.2261–1.9879	0/1	0.0000–0.0000
Buzău (BZ)	11/1406	0.7608–0.8039	0/97	0.00000–0.00000	10/2333	0.4086–0.4487	8/12	39.9944–93.3389
Total	127/131,759	0.0948–0.0980	5/23,748	0.01923–0.02288	156/10,695	1.2314–1.6858	25/68	25.3044–48.2250

C.I. 95% (Confidence Interval; α = 0.05 was considered as statistically significant).

**Table 3:** Geographical prevalence of *T. spiralis* in pigs, horses, wild boars and bears, in North-Eastern Romania.

County	Pig		Horse		Wild Boar		Bear	
	Positive/ Tested	C.I. [a–b]	Positive/ Tested	C.I. [a–b]	Positive/ Tested	C.I. [a–b]	Positive/ Tested	C.I. [a–b]
Suceava (SV)	0/1109	0.0000–0.0000	5/18,407	0.00003–0.00051	20/34	42.2804–75.3666	4/6	28.9379–100.000
Botoșani (BT)	1/12,308	0.0000–0.0002	0/5244	0.00000–0.00000	1/145	0.6144–0.7650	0	0.00000–0.00000
Neamț (NT)	1/107,675	0.00000–0.00003	0/0	0.00000–0.00000	0/211	0.0000–0.0000	0	0.00000–0.00000
Iași (IS)	0/183	0.0000–0.0000	0/0	0.00000–0.00000	15/500	1.5047–4.4956	0	0.00000–0.00000
Bacău (BC)	3/125	0.0000–0.0508	0/0	0.00000–0.00000	56/67	74.7119–92.4523	5/6	53.5072–100.0000
Vaslui (VS)	12/65	0.0903–0.2789	0/0	0.00000–0.00000	0/338	0.0000–0.0000	0	0.00000–0.00000
Vrancea (VN)	3/7825	0.0000–0.0008	0/0	0.00000–0.00000	11/19	35.6940–80.0955	2/5	0.0000–82.9414
Galați (GL)	96/1063	0.0731–0.1075	0/0	0.00000–0.00000	6/542	0.2261–1.9879	0	0.00000–0.00000
Buzău (BZ)	11/1406	0.0032–0.0124	0/97	0.00000–0.00000	10/2333	0.1635–06937	5/8	28.9520–96.0480
Total	127/131,759	0.0008–0.0011	5/23,748	0.00003–0.00040	119/4189	2.3377–3.3439	16/25	45.1840–82.8160

**Table 4:** Geographical prevalence of *T. britovi* in wild boar and bears in North-Eastern Romania.

County.	Wild Boar		Bears	
	Positive/ Tested	C.I. [a–b]	Positive/ Tested	C.I. [a–b]
Suceava (SV)	14/34	24.6334–57.7196	2/6	0.0000–71.0536
Bacău (BC)	11/67	7.5477–25.2881	1/6	0.0000–46.4871
Vrancea (VN)	8/19	19.9045–64.3060	3/5	17.0586–100.000
Buzău (BZ)	0/2333	0.0000–0.0000	3/8	3.9520–71.0480
Vaslui (VS)	4/338	0.0306–2.3363	0	0.0000–0.0000
Total	37/2791	0.9014–1.7500	9/25	17.1840–54.8160

## Data Availability

Not applicable.
